# Comparison of Nutrition among Female Floorball Players of Extra-Class Teams from Poland and the Czech Republic during the Preparation Period for the League Season

**DOI:** 10.3390/nu16040544

**Published:** 2024-02-16

**Authors:** Agnieszka Białek-Dratwa, Zuzanna Krzywak, Wiktoria Staśkiewicz-Bartecka, Jiří Velecký, Artur Cirocki, Mateusz Grajek, Oskar Kowalski

**Affiliations:** 1 Department of Human Nutrition, Department of Dietetics, Faculty of Health Sciences in Bytom, Medical University of Silesia in Katowice, ul. Jordana 19, 41-808 Zabrze, Poland; okowalski@sum.edu.pl; 2 SUS Travel Jedynka Trzebiatów, ul.Kamieniecka 16, 72-320 Trzebiatów, Poland; 3 Department of Food Technology and Quality Evaluation, Department of Dietetics, Faculty of Health Sciences in Bytom, Medical University of Silesia in Katowice, ul. Jordana 19, 41-808 Zabrze, Poland; 4 1. SC Tempish Vitkovice (Ostrava), 708 00 Ostrava, Czech Republic; 5 Department of Public Health, Department of Public Health Policy, Faculty of Health Sciences in Bytom, Medical University of Silesia in Katowice, ul. Piekarska 18, 41-902 Bytom, Poland; mgrajek@sum.edu.pl

**Keywords:** floorball, diet, professional athletes, eating habits

## Abstract

The study aimed to assess the frequency of food intake and to compare the consumption of female extramural players training floorball in Poland and the Czech Republic during the preparation period for the league season. In total, 43 players training floorball in senior clubs participated in the study, including 21 from the Polish and 22 from the Czech clubs. The research tool was based on the standardised questionnaire for the Examination of Eating Behaviours and Opinions on Food and Nutrition (QEB). The study also analysed body composition using the Bioelectrical Impedance Analysis (BIA) method, and the research tool was a TANITA MC-780 S MA body composition analyser (Tanita Corporation, Tokyo, Japan). The Polish women’s floorball players had lower results in body fat percentage (FM) and muscle mass (MM) than the Czech team. The mean FM in the players of the Polish team was 18.6% ± 5.4, and the mean MM was 45.8 kg ± 4.2. In the Czech team players, these figures were 19.8% ± 5.4 and 47.8 kg ± 4.2. Despite the similar value of mean BMI in both teams, the highest BMI in the case of female athletes from Poland (17.7), indicating underweight, and the highest BMI in female athletes from the Czech Republic (26.9), indicating overweight, were significant. The study showed differences in both body composition analysis and dietary patterns of the Czech and Polish players. The Czech women’s floorball players had a higher muscle mass and body fat percentage than Polish floorball players. Furthermore, differences in diet were observed among the players of the Czech team compared to the players of the Polish team. The Czech women’s floorball players consumed a slightly higher amount of healthier products, such as whole-grain products. The Polish players took in more meat, processed products and fruit juices. This study is one of the first to assess the nutrition of those involved in floorball. There is a need for further research that focuses on the specifics of the discipline, the exercise capacity of the players and points during the season that require nutritional support. This knowledge would help develop effective nutritional strategies and plan and implement appropriate nutrition education for this group of athletes.

## 1. Introduction

Nutrition is integral at every stage of an athlete’s career. It significantly impacts the performance and efficiency of athletes and recovery between training sessions [1]. A proper diet is an essential component of training plans [2]. A properly balanced nutritional plan will be associated with increased performance during training and matches and, consequently, better sporting results [1]. Furthermore, it can significantly reduce the risk of infections, [3] injuries or injuries resulting from overtraining and insufficient energy availability. Due to different anthropometric characteristics, each athlete should be treated individually, and the athlete’s diet should be adapted to the unique nutritional needs associated with physical activity [4].

Women who participate in team sports have different dietary needs than men. We chose football and indoor football to establish the principles of nutrition and the impact of nutrition on performance in this sport because, regrettably, there is currently no research on diets and nutrition advice for women playing floorball.

Each athlete has his or her energy requirement, which is determined by the sports goals, basal metabolism, thermogenesis, individual physical activity of the athlete and the league season, as well as sudden injuries and breaks in the preparation period [5,6]. Women may need less energy because of their smaller bodies and less intense exercise [7]. In order to approximate the energy needs of female football players, it is essential to assess their energy expenditure, which is heavily influenced by factors such as competition level, football position, style of play, and training intensity [1]. For this reason, figuring out each player’s specific energy needs requires a customized strategy. It may be necessary to overestimate or underestimate each player’s unique needs to adhere to the guidelines in the literature [1]. High-measurement precision devices, such as motion sensors like accelerometers and pedometers, should be used to estimate energy requirements [8,9,10]. Energy intake should be minimal in fat and optimally correlated with the athlete’s physical condition and lean body mass. To avoid the negative impacts of low energy availability on production and health, the daily energy content of the diet should not be lower than 30 kcal/kg lean body mass [1].

An appropriate energy value of the dietary ration is the consequence of consuming the proper amounts of the macronutrients—fats, carbs, and proteins. Macronutrients play specialized roles in the body of athletes. Thus, optimizing performance and increasing the likelihood of winning in sporting events is directly related to having an adequate supply of them [1].

For a moderate-length, low-intensity training program, female football players should consume 5–7 g of carbohydrates per kilogram of body weight per day; for moderate-to-heavy endurance training, they should consume 7–12 g per kilogram per day [1].

Before a workout or game, consuming carbs for a predetermined amount of time boosts glycogen storage, stabilizes blood sugar, and preserves glycogen in the liver and muscles. For activities lasting longer than 90 min, an effective dose of 10–12 g CHO/kg body weight/day every 24 h for 36–48 h is recommended [11].

An athlete’s diet must include proteins since they are necessary for regulating muscle protein synthesis, controlling body weight, promoting growth, and recovering after exercise, among other processes. Amino acids and proteins are essential for team sports players to function well [12]. The American College of Sports Medicine, the Dietitians of Canada, and the Academy of Nutrition and Dietetics all recommend that an individual’s daily protein intake should be between 1.2 and 2.0 g/kg body weight. Higher intakes are necessary for short-term training intensification, and diet-based energy intake should be decreased [13]. Protein recommendations are appropriate for male and female football coaches, even if most studies and recommendations focus on men or are directed towards all athletes [14].

Fats are an extra energy source and a crucial part of cell walls. They are a good source of fat-soluble vitamins, one of which is vitamin D, which is particularly noteworthy. It is advised that athletes avoid consuming less than 20% of their diet’s caloric content as fat for an extended period, as this may decrease vital fatty acids and fat-soluble vitamins [13].

For female athletes and athletes in general, iron-containing goods are vital components of their diets. In addition to impairing adaptation to aerobic training, increasing muscle fatigue, and lowering energy output during submaximal exercise, iron deficiency anaemia can lead to decreased performance [15]. Iron deficiency among athletes is especially dangerous for women [15,16,17]. This is partially because this micronutrient was sometimes lost [17]. It is noteworthy that female athletes may require up to 70% more iron than what is recommended by the EAR [13].

The detrimental effects of calcium and vitamin D deficiency are hazardous for athletes, especially women athletes. According to studies, young women who engage in intense activity risk having their bone mineral growth slowed [18]. As discussed later, menstruation abnormalities or limited energy availability may contribute to reduced bone mineral density. Calcium and vitamin D deficiency leads to a decrease in bone mass and a higher chance of fractures. There may lead to a temporary ban from physical activity, potentially irreversible health damage, and the inability to resume regular exercise. The athlete’s fitness will significantly decline, and they will be unable to play well for a while. This will affect the athlete’s entire career and other aspects of life (e.g., economics).

This research among female floorball players is one of the first studies to consider body composition analysis and eating behaviour in this group of women.

This study aimed to assess the frequency of food intake and to compare the consumption of female extramural players training floorball in Poland and the Czech Republic during the preparation period for the league season.

## 2. Materials and Methods

### 2.1. Differences between the Polish and Czech League

Floorball is a team sport still developing worldwide, often associated with ice hockey, but without ice and skates. Players use a lightweight plastic ball and sticks made of carbon fibre to play the game. A floorball match is played between teams of up to 20 players, with 5 field players and a goalkeeper simultaneously playing on the field. The match consists of three 20 min periods, and the team that scores the most goals is the winner [19,20].

Due to the high pace of the game, floorball is a very demanding sport. Paradoxically, although it is considered a non-contact sport, there are many clashes between players. Rapid direction changes, accelerations and decelerations mean that players must display motor skills and physical strength [20].

Since founding the International Floorball Federation (IFF) in 1986, floorball has rapidly evolved into one of the fastest-growing sports [21]. According to data from 2020, the IFF has more than 350,000 registered players from 71 countries. Of these, precisely 41,404 are in the Czech Republic, while only 2185 are in Poland [22]. There are also differences between the two countries in the year they joined the IFF; the Czechs became members of the International Floorball Federation in 1993 [23], while Poland only became members six years later [24]. Furthermore, IFF data show that 103 clubs are registered in Poland [24], which cannot compare to the number of clubs in the Czech Republic (411 clubs) [23].

In both countries, there is competition among both men and women. In the Czech Republic, there are 112 men’s clubs and 37 women’s clubs [25], while in Poland, the numbers are 16 and 6, respectively [26]. The floorball tournament, in which players compete for the title of champion of Poland and the Czech Republic, is held annually in both countries [25,26].

In the Czech Republic, there are two women’s leagues, the Ekstraliga (the highest competition league) with 12 teams and the 1st league with 25 teams. Although the length of the competition season in the two countries does not differ much, the course of the season is different. Currently in the Czech Republic, the competition season starts in September and ends in April of the following year. It is an intensive eight months in which the players play an average of 22 matches in the regular round (teams play matches on an “every-man-for-all” basis, after which a certain number of them, with the highest number of points, advance to the play-off phase) and a maximum of 15 in the play-off phase (the decisive phase of the competition, ending with the final match, in which the champion team is determined). This results in a total of 37 matches, an average of 1–2 per week. During the playing season, in addition to the matches, the players are indoors about 3 times a week, engage in stick training (the training is then endurance/strength training) and train in the gym 1–2 times a week (strength/circuit training), which last on average 1.5 h. In addition, during the preparatory season, which lasts from May to August, the amount of indoor training decreases in favour of training performed on a treadmill or outdoors (endurance training) and strength training [25].

The season in Poland differs from that in the Czech Republic. According to 2022, it runs from September to May of the following year. Only one women’s league (Ekstraliga) is in Poland, with only six teams competing. Over nine months, the players play ten matches in the regular round and a maximum of eight in the play-off phase. This is a total of 18 matches, which is half as many as in the Czech Republic. According to calculations, considering the season’s length, women’s floorball players in Poland play an average of one match every 2 weeks. During the playing season, floorball players perform indoor training (endurance training) on average 3 times a week, lasting approximately 1.5–2 h, and strength training is primarily not performed. The preparatory season, in which the whole team participates, mostly starts a few weeks before the games start and is mainly based on indoor and cross-country training (which is endurance in nature) [26].

In total, 43 players training floorball in senior clubs participated in the study, including 21 from the Polish and 22 from the Czech clubs. All players currently training with both teams and actively playing league matches participated in the study. The study was conducted in August and September 2021—during the preparatory period. The preparatory period for the league season was chosen to conduct a study on eating habits and body mass composition for several reasons. During the preparation period, training is strictly set and focused on developing overall physical form. The players usually have regular training sessions, which makes it easier to monitor the impact of diet on their performance. During the preparation period, female athletes often work on building muscle mass, which can affect their body composition. Testing during the preparatory period provides coaches with important information regarding the physical condition of female athletes before the season begins. The preparatory period is the optimal time for testing, as it provides key information to optimise the physical form of players before the league season and allows for possible adjustments to nutritional habits.

Inclusion criteria for the study were being an active player of the team included in the study (the player must have signed a professional contract), being above 18 years of age, actively preparing for the 2021/2022 season, and having knowledge of Polish or Czech. The exclusion criteria for the study were an injury excluding for more than four weeks from active preparation for the 2021/22 season and age below 18 years. After considering the inclusion and exclusion criteria, 40 players qualified for the study: 20 from a Polish club and 20 from a Czech club. The study was conducted by the World Medical Association’s Helsinki Declaration. The Bioethics Committee of the Silesian Medical University in Katowice evaluated and approved the study protocol (PCN/0022/KB/68/I/20). All female athletes studied gave written informed consent to participate in the study. The study was conducted in Poland and the Czech Republic.

### 2.2. Characteristics of the Study Group

The study was conducted among female players practising floorball: 20 represented the Polish club “Extraliga polska w unihokeju kobiet”, while another 20 represented the Czech club “Extraliga žen ve florbale”. All players on the Polish team were of Polish nationality, while the Czech team had 2 Polish, 4 Slovakian and 14 Czech players. Every player was over the age of 18. The average age of the Polish women’s floorball players was 22.0 years, while that of the Czech team was 21.8 years.

The majority of players on both the Polish and Czech teams were in the attacking position. The players included 10 on the Polish team (50%) and 12 (60%) on the Czech team. The fewest players in the study took the goalkeeper position. There were 4 (20%) goalkeepers among the Polish floorball players, while none of the Czech players were included in the study.

### 2.3. Research Tool

The research tool was based on the standardised questionnaire for the Examination of Eating Behaviours and Opinions on Food and Nutrition (QEB) [27,28,29]. The QEB questionnaire consists of sections including the study of eating habits, frequency of food intake, opinions on food and nutrition, self-assessment of diet, knowledge of nutrition and its source, and personal data, which was extended to include information on field position or frequency of training. The survey was conducted by means of a face-to-face interview with a qualified person. During the interview, each athlete was free to answer the questions asked.

In this publication, the first and second parts of the questionnaire on food frequency were used in detail, as well as the typical composition of participants’ meals and the frequency of consumption of selected foods (frequency of consumption was assessed on the following scale: “never”, “1–3 times a month”, “once a week”, “several times a week”, “once a day”, “several times a day”).

### 2.4. Body Composition Analysis Procedure

The study also analysed body composition using the Bioelectrical Impedance Analysis (BIA) method, and the research tool was a TANITA MC-780 S MA body composition analyser (Tanita Corporation, Tokyo, Japan).

The equipment used in the study is approved for medical use and meets NAWI CLASS III standards for scales used for medical measurements. The analyser has the EU certification CE0122. Regarding medical devices, it meets the requirements of the MDD 93/42/EEC (Medical Device Directive). The test subject’s torso, arms and legs were individually measured for impedance at different frequencies (multifrequency—5 kHz/50 kHz/250 kHz) using an octapolar eight-point touch electrode system [30]. 

The athletes taking part in the study were given guidelines according to the information indicated by the manufacturer not to consume alcohol, to avoid caffeine and heavy physical exertion and to come to the study on an empty stomach. In addition, they were informed of the contraindications to the study. During the measurement, the subjects assumed a standing position and uncovered their feet and hands for accurate measurement. The study was performed in September 2022 in the morning. Body composition analysis was performed on each athlete on three occasions over a period of 5 min consecutively. And then the average value of all three measurements was calculated.

Parameters assessed were body weight, height, age, body mass index (BMI—body mass index), basal metabolic rate (BMR—basal metabolic rate), total body water (TBW—total body water), visceral fat rating (VFR—visceral fat rating), mineralised bone mass and percentage body fat (FM—fat mass) and muscle mass (MM—muscle mass).

### 2.5. Statistical Analysis

Microsoft Office Word 365, Microsoft Office Excel 365 and Statistica 13 were used to analyse the collected data. The results were collected in table form using the following statistical analyses: arithmetic mean ($\bar{x}$) and standard deviation (SD). The Shapiro–Wilk test assessed the distribution of quantitative variables such as anthropometric measurements. The distribution of anthropometric measurements was not expected; therefore, the Mann–Whitney U test was used to compare the mean values of anthropometric parameters between the team from Poland and the Czech Republic. The Chi^2^ test was used to assess differences in the intake of individual foodstuffs in the two study teams; a significance level of *p* = 0.05 was assumed.

## 3. Results

### 3.1. Characteristics of the Study Group

Table 1 shows the characteristics of the studied group of female floorball players from the Polish and Czech teams. The mean body mass of the female floorball players from the Polish team was lower (57.7 kg ± 6.3) compared to the subjects from the Czech team (63.1 kg ± 7.4). The mean height in both groups was 166.0 ± 6.2 cm vs. 166.8 ± 5.0 cm. The mean BMR of the Polish players was 1439.1 ± 101.4 kcal, while that of the Czech players was 1520.8 ± 125.4 kcal. The lower results of the female floorball players on the Polish team were recorded in percentage body fat (FM) and muscle mass (MM). The mean FM in the players of the Polish team was 18.6% ± 5.4, and the mean MM was 45.8 kg ± 4.2. In the Czech team players, these figures were 19.8% ± 5.4 and 47.8 kg ± 4.2. Despite the similar value of mean BMI in both teams, the lowest BMI in the case of female athletes from Poland (17.7), indicating underweight, and the highest BMI in female athletes from the Czech Republic (26.9), indicating overweight, were significant. The body composition analysis data showed no differences for body height, age, visceral fat ratio (VFR) or mineralised bone mass.

The study assessed the use of diets by female athletes and sources of knowledge on proper nutrition in sports (Table 2). The vast majority of female athletes of the Polish club had never taken the advice of a nutritionist (95%), and only one person (5%) during the study had not recently but had done so in the past. In the case of the Czech team, more people had sought the advice of a dietician in the past, including four people (20%), while as in the case of the respondents from the Polish team, the majority of people (80%) were players who had never used dietetic services. The most common source of acquiring knowledge on proper nutrition in both teams was the Internet. Among the players of the Polish club, 14 (70%) used it, and among the Czech club, 12 (60%). The fewest respondents from Poland used coaches/trainers (5%), scientific literature (5%) and other (5%) sources as those they could use to gain knowledge on correct nutrition. In contrast, the rarest source among Czechs was scientific literature (5%). Among Polish league players, the majority train 2–4 times a week, while Czech league players train 5 times a week or more (*p* = 0.000). Female players of the Polish team most often train endurance, while female players of the Czech team train strength and endurance (*p* = 0.0428). Strength training is more common among Czech female athletes than Polish female athletes (*p* = 0.000). When considering the number of training hours, both female athletes of the Polish and Czech teams spend 1–3 h per day training (*p* = 0.2347).

### 3.2. Eating Habits among Female Floorball Players Surveyed

Table 3 shows the results on selected issues related to eating habits. The type of meal most frequently consumed in both teams was home-cooked meals. In both cases, the number who consumed them represented 100% of all players. Polish and Czech team players consumed most of the meals they made themselves. In both teams, the number was 19 players each, which accounted for 95%.

The respondents were asked whether they adapted their diet to their physical activity. Half (50%) of the female athletes from the Polish team do not know if they do, and only three (5%) answered yes. In the case of the Czech team, the vast majority (75%) were respondents who answered ‘yes’, and only 10% denied it. Most female floorball players from Poland eat the same in the off-season as during the sports season (80%). In the case of Czech players, the majority eat the same during the season as during the off-season (45%), while there are almost twice as many of them as Polish women. In addition, 35% eat less in the off-season than during the season, and 20% eat more.

Most of the female athletes of the Polish team (50%) usually consume three meals a day, while the minority, only 5% of the respondents, eat two meals or five meals or more. Among respondents from the Czech Republic, the highest proportion of female athletes consumed four meals (55%) per day and the lowest consumed two meals (5%).

In both teams, respondents answering the question about eating at fixed times of the day mostly answered that they do so, but only for certain meals. This figure was 65% for the Polish athletes and 70% for the Czech athletes. Among both teams, only one person (5%), the Czech female athlete, eats all meals at fixed times of the day.

The respondents were asked how often they snack between meals. The most significant number of female athletes of the Polish team snack several times a week (65%). The minority snack 1–3 times a week (5%). Similarly to Polish female athletes, most female athletes in the Czech team also snack several times a week (60%). On the other hand, the smallest number of them snack several times a day (5%). Among the female athletes of the Polish club, the most frequent foods snacked on between meals were fruit (15 athletes—75%) and sweet snacks (15 athletes—75%), while the least frequent foods were nuts, almonds, seeds and pips (4 athletes 20%). None of the respondents marked vegetables as a food they usually eat between meals. For Czech club athletes, the responses were very similar. The most reported snack was fruit (16 athletes—80%), followed by sweet snacks (14 athletes—70%), and the fewest responses were for vegetables (3 athletes—6%).

The respondents were asked whether they sweetened hot drinks such as coffee and tea. The most significant number of female athletes (65%) sweeten their drinks with one teaspoon of sugar or honey. The fewest (10%) sweeten with two or more teaspoons. The results of the Czech team were very similar to those of the Polish team. Most of the players surveyed sweetened drinks with one teaspoon (70%), while the fewest were those who sweetened drinks with two or more teaspoons of sugar or honey (5%). The female athletes of both teams were asked whether they added salt to prepared food and sandwiches at the table. Most respondents of the Polish team salt their dishes, but only sometimes (60%). The rest (40%) do not. In the case of Czech female athletes, most did not salt their food (50%), and slightly fewer did so only sometimes (40%). Only 10% of all Czech women’s floorball players salt most of their food.

The study also included a selection of dairy products with different fat contents. The Polish subjects overwhelmingly consumed milk and milk drinks with a standard fat content (14 athletes, 70%). In total, 30% of them consumed dairy products with a reduced fat content. The Czech athletes had very different results, as milk products with a reduced fat content accounted for the majority (14 athletes 70%) of responses, and only 30% of respondents consumed milk and milk drinks with a standard fat content.

The preparation method of meat dishes was also assessed, considering heat treatment. In the case of the players of the Polish team, the vast majority of responses were for baked (14 players) and fried dishes (14 players). The smallest number of answers concerned grilled dishes (three athletes). In the Czech team, frying was the most frequently chosen thermal treatment (11 female athletes), and stewing was the least frequently chosen thermal treatment (4 female athletes). In addition, two athletes from the Czech team indicated that they do not eat meat.

In the Polish and Czech teams, butter was the most commonly used fat for spreading bread. This was reported by 12 (60%) players of the Polish team and 11 (55%) of the Czech team. In both clubs, the most frequently used fat for frying food was vegetable oil (including olive oil). Among the Polish players, this number was 18 (90%), while among the Czech players, it was 17 (85%). The remainder of the respondents used different fats.

There were no differences in product consumption between the two clubs. There were slight differences in the consumption of 7 out of 34 products. Table 4, Table 5, Table 6 and Table 7 show the results on the frequency of consumption of each food and drink group (Table 4, Table 5, Table 6 and Table 7). The Czech players were slightly more likely to consume wholemeal bread (several times a week—40%, once a day—15%), coarse oats, oatmeal and wholemeal pasta (several times a week—55%, once a day—5%) and isotonic drinks (1–3 times a month—50%, once a week—5%, several times a week—30%). Polish players, on the other hand, were more likely to consume products such as fried foods (several times a week—55%, once a day—10%), cold cuts, sausages and wieners (several times a week—35%, once a day—10%, several times a day—10%), fruit juices (several times a week—35%, once a day—5%) and water (several times a day—95%).

The study analysed whether the amount of water consumption on a training day differed between the two teams. Most Polish players consume 1–1.5 litre of water (n = 9, 45%), with five players each consuming 1.5–2 litre and more than 2 litre. In contrast, in Czech women’s floorball players, it is more than 2 litre (n = 10, 50%), and 25% of players drink 1.5–2 litre (n = 5).

## 4. Discussion

Even though floorball is an ever-evolving sport of continuing interest, minimal research has been carried out. This study is one of the first to address the nutrition of this group of athletes. It analyses the results of a BIA test and evaluates the diets of female floorball players from the Polish and Czech teams. Due to the scarcity of studies on floorball, these results were compared to players of team sports or other sports similar to floorball in their characteristics. These include, among others, hockey, football, handball or basketball, which are characterised by rapid changes in running direction, accelerations and decelerations. In addition, both physical and mental strength are essential to them.

Many factors influence food choices. These include the price of products, individual taste, culture, religion or family diet. In addition, physiological factors related to feelings of hunger and satiety, allergies, food intolerances and gastrointestinal complaints, lifestyle factors and psychological factors affect the desire to look after one’s figure and sporting form [31]. The pyramid of healthy eating for athletes indicates that animal fats should be limited in the diet, vegetable fats should be consumed daily, proper hydration should be ensured by consuming unsweetened beverages, fast-food should be avoided, and salty and sugary snacks should be limited. In addition, it is advisable to eat an adequate number of regular meals (a minimum of 3 per day), to consume 2–3 portions of vegetables and 1–2 portions of fruit and to ensure a good supply of milk and dairy products (2 per day). Attention is also drawn to the consumption of whole-grain cereal products twice a day and ensuring an adequate fish intake (1–2 times a week) [32].

The study showed a slight difference in the intake of selected foodstuffs between the players on the Polish and Czech teams. Female athletes on the Czech team were more likely to consume whole-grain products and isotonic drinks, whereas female athletes on the Polish team consumed fried foods, processed meat products such as sausages or cold cuts, and fruit juices. In addition, it has been shown that most Czech players consume milk and reduced-fat dairy products, in contrast to the Třeboň players. A more significant number of Polish female floorball players consume milk and milk products with a standard fat content. The results from the questionnaire also indicate that the players from the Czech team are more likely to adapt their diet to their physical activity.

Despite the insignificant difference, such nutritional choices may have been because the players of the Czech team had received advice from a dietician more often in the past and that coaches were a more frequent source of knowledge on proper nutrition compared to the female floorball players of the Polish team. In addition, the players of the Czech team mostly rated their health status as better than that of their peers (50%) and their nutritional knowledge as good (45%), in contrast to the Polish players, for whom a percentage of these responses was lower (25% and 40%, respectively). More sound knowledge may have translated into their nutrition choices. There are many studies on athletes’ food intake and eating behaviour [32,33,34,35,36]. However, there is a need for more thorough research on a group such as university hockey players.

Hydration in a group of athletes is just as important as adequate food intake. Too little water in the body of athletes can lead to dehydration, which is associated with many consequences. Among other things, it will deteriorate their performance and endurance and weaken the body [37]. Research suggests that athletes do not follow recommendations for adequate water supply [38,39], and a study by Witold Kozirok et al. [38] found that several factors, such as the amount and timing of training, the sport and the age of the athletes, influences fluid intake. The intake frequency survey showed more frequent water intake by the Czech athletes, while in the survey question on the amount of water consumed on a training day, the athletes from the Czech team took in more water. Considering the results, the Czech women’s floorball players care about hydration only during the day when training or a match takes place.

Because of the mistakes that female floorballers make, there is a need for more research focused on the sport and education of this group, which will address the critical issues of the importance of adhering to nutritional recommendations and adequate fluid supply [40].

However, the study has limitations as the study group only included two clubs, one in each country, so an extension of the study seems justified. In addition, the food frequency questionnaire is not a tool that reflects the daily diet of female athletes without error. However, according to the current literature review, this is the first study evaluating female floorball players’ diet and body mass composition. Moreover, the study compares the results obtained from two countries, providing innovative and valuable results with comparative value for further research. This study is one of the first to target the nutrition of athletes in this sport. There is a need for further research that focuses on the specifics of floorball, the female players and their nutrition. This knowledge would help to develop dietary plans for this group of athletes, implement appropriate nutrition education and raise awareness of their health status.

The nutritional recommendations for female floorball players presented in our article have some limitations. The main limitation in terms of providing concise recommendations for female floorball coaches is the lack of studies that included female floorball players as a study group.

## 5. Conclusions

The study showed differences in both body composition analysis and dietary patterns of the Czech and Polish players. The Czech women’s floorball players had a higher muscle mass and body fat percentage than Polish floorball players. Furthermore, differences in diet were observed among the players of the Czech team compared to the players of the Polish team. The Czech women’s floorball players consumed a slightly higher amount of healthier products, such as whole-grain products. On the other hand, the Polish players took in more meat, processed products and fruit juices.

This study is one of the first to assess the nutrition of those involved in floorball. There is a need for further research that focuses on the specifics of the discipline, the exercise capacity of the players and points during the season that require nutritional support. This knowledge would help develop effective nutritional strategies and plan and implement appropriate nutrition education for this group of athletes.

### Practical Implications

The following nutrition rules are recommended for female floorball players:

1. The energy needs of floorball players are individual and an individual assessment of energy expenditure is necessary, which is strongly influenced by factors such as the level of competition, the position of the player, the style of play and the intensity and length of training. Such assessments can be carried out using motion sensors such as accelerometers and pedometers or more complex devices to assess daily energy requirements.
2. For a moderate-length, low-intensity training programme, floorball players should consume 5–7 g of carbohydrate per kilogram of body weight per day; for moderate or heavy endurance training, they should consume 7–12 g per kilogram per day.
3. Before training or a match, consuming carbohydrates for a set period of time increases glycogen storage, stabilises blood sugar levels and preserves glycogen in the liver and muscles. For activities lasting longer than 90 min, an effective dose of 10–12 g CHO/kg body weight/day every 24 h for 36–48 h is recommended.
4. It is recommended that a specially formulated sports nutrition supplement is consumed immediately after training, followed by a more substantial carbohydrate-rich meal to help athletes replenish their carbohydrate stores and reach their individual target intake after days of intense exercise.
5. A daily protein intake of 1.2 to 2.0 g/kg body weight is recommended. A higher intake is necessary for short-term intensification of training, especially strength training.
6. Adequate hydration of the athlete is important. Players are recommended to drink 5–7 mL/kg body weight four hours before training and an additional 3–5 mL/kg body weight two hours before the start of exercise in cases of no urine or very dark urine. Hydration during training depends on the type and intensity of exercise. It is recommended to drink 1.2–1.5 L of fluids for every kilogram of body weight lost during training or a match. The players’ sweating rate, weight loss and sweat sodium concentration ([Na]+) are higher during matches than during training. The high inter-athlete variability highlights the need for individualised hydration strategies to avoid negative consequences on performance and health.

However, there have been no studies specifically addressing the needs of female floorball players. Most recommendations for this group are based on those for female football players. Further research is needed on the nutritional needs of women playing this sport and the risk of adverse effects resulting from an inappropriate diet.

## Figures and Tables

**Table 1:** Physical characteristics and variables of body composition including the team’s country (POL—Polish team, CZ—Czech team).

	POL N = 20 (100%)	CZ N = 20 (100%)	*p* Value
Age [years]	22.0 ± 3.1	21.8 ± 3.5	ns
Body weight [kg]	57.7 ± 6.3	63.1 ± 7.4	ns
Body height [cm]	166.0 ± 6.2	166.8 ± 5.0	ns
BMI [kg/m^2^]	21.0 ± 2.1	22.7 ± 2.0	ns
BMR [kcal]	1439.1 ± 101.4	1520.8 ± 125.4	ns
FM [%].	18.6 ± 5.4	19.8 ± 5.4	ns
TBW [%]	61.4 ± 3.4	60.0 ± 4.0	ns
VFR	1 ± 0	1 ± 0	ns
Mineralised bone mass [kg]	2.4 ± 0.2	2.5 ± 0.2	ns
MM [kg]	45.8 ± 4.2	47.8 ± 4.2	ns
**Position on the pitch**			
Defender	6 (30%)	8 (40%)	ns
Attacker	10 (50%)	12 (60%)	
Goalkeeper	4 (20%)	0 (0%)	
	**Frequency of training**		
2–4 times a week	16 (80%)	0 (0%)	*p* = 0.000
5 times a week	4 (20%)	10 (50%)	
6 times a week and more	0 (0%)	10 (50%)	
	**Type of training**		
Circuit (strength + endurance)	4 (20%)	11 (55%)	*p* = 0.0428
Strength (resistance)	1 (5%)	0 (0%)	
Endurance (conditioning)	15 (75%)	9 (45%)	
	**Frequency of strength training**		
Once a week	2 (10%)	8 (40%)	*p* = 0.000
2–3 times a week	3 (15%)	12 (60%)	
I do not train at a gym	15 (75%)	0 (0%)	
	**Frequency of training in hours**		
1 h	1 (5%)	0 (0%)	*p* = 0.2347
1–3 h	19 (95%)	20 (100%)	

ns—not statistically significant.

**Table 2:** Use of diet by female athletes in the surveyed sports clubs and sources of knowledge on proper nutrition (POL—Polish team, CZ—Czech team).

	POL N = 20 (100%)	CZ N = 20 (100%)	*p* Value
**Application of the diet**			
Not	20 (100%)	20 (100%)	ns
Yes	0 (0%)	0 (0%)	
**Taking advice from a nutritionist**			
No, I have never	19 (95%)	16 (80%)	ns
I do not currently, but I have in the past	1 (5%)	4 (20%)	
Yes, I am currently under the care of a nutritionist	0 (0%)	0 (0%)	
**Source of knowledge on** **proper nutrition**			
Nutritionist	0 (0%)	0 (0%)	ns
Coach(es)	1 (5%)	5 (25%)	
Internet	14 (70%)	12 (60%)	
Scientific literature	1 (5%)	1 (5%)	
From other players	3 (15%)	2 (10%)	
Other	1 (5%)	0 (0%)	

ns—not statistically significant.

**Table 3:** Selected aspects of nutritional habits among female athletes in surveyed sports clubs (POL—Polish team, CZ—Czech team).

	POL N = 20 (100%)	CZ N = 20 (100%)	*p* Value
**Type of meal**			
Home-cooked meals	20 (100%)	20 (100%)	ns
Catering	0 (0%)	0 (0%)	
Meals prepared in town	0 (0%)	0 (0%)	
Store-bought ready meals	0 (0%)	0 (0%)	
Other	0 (0%)	0 (0%)	
**Are the meals made independently?**			
Yes	19 (95%)	19 (95%)	ns
Not	1 (5%)	1 (5%)	
**Adapting diet to physical activity**			
Yes	3 (5%)	15 (75%)	ns
Not	7 (35%)	2 (10%)	
I don’t know	10 (50%)	3 (15%)	
**Does he/she eat the same in the off-season as during it?**			
Yes	16 (80%)	9 (45%)	p = 0.00040
No, I eat less than during the season	3 (15%)	7 (35%)	
No, I eat more than during the season	1 (5%)	4 (20%)	
**Number of meals typically consumed per day**			
2 meals	1 (5%)	1 (5%)	ns
3 meals	10 (50%)	6 (30%)	
4 meals	8 (40%)	11 (55%)	
5 meals or more	1 (5%)	2 (10%)	
**Eating at regular times of the day**			
Not	7 (35%)	5 (25%)	ns
Yes, but only sometimes	13 (65%)	14 (70%)	
Yes, all the time	0 (0%)	1 (5%)	
**Frequency of snacking between meals**			
1–3 times per month	1 (5%)	0 (0%)	ns
Once a week	2 (10%)	1 (5%)	
Several times a week	13 (65%)	12 (60%)	
Once a day	4 (20%)	6 (30%)	
Several times a day	0 (0%)	1 (5%)	
**Sweetening of hot drinks**			
Not	5 (25%)	5 (25%)	ns
Yes, with one teaspoon of sugar or honey	13 (65%)	14 (70%)	
Yes, with two or more teaspoons of sugar or honey	2 (10%)	1 (5%)	
**Adding salt to prepared foods and sandwiches at the table**			
No	8 (40%)	10 (50%)	ns
Yes, but only sometimes	12 (60%)	8 (40%)	
Yes, I add salt to most dishes	0 (0%)	2 (10%)	

ns—not statistically significant.

**Table 4:** Frequency of consumption of traditional foodstuffs by female athletes of both study groups (POL—Polish team, CZ—Czech team).

Products	Club	Frequency of Consumption N (%)						*p* Value
		Never	1–3 Times per Month	Once a Week	Several Times a Week	Once a Day	Several Times a Day	
White bread	POL	0 (0%)	1 (5%)	3 (15%)	8 (40%)	6 (30%)	2 (10%)	ns
	CZ	0 (0%)	1 (5%)	3 (15%)	11 (55%)	3 (15%)	2 (10%)	
Wholemeal bread	POL	0 (0%)	10 (50%)	7 (35%)	3 (15%)	0 (0%)	0 (0%)	*p* = 0.046
	CZ	1 (5%)	4 (20%)	4 (20%)	8 (40%)	3 (15%)	0 (0%)	
White rice, plain pasta or small oats	POL	0 (0%)	0 (0%)	3 (15%)	17 (85%)	0 (0%)	0 (0%)	ns
	CZ	0 (0%)	1 (5%)	1 (5%)	17 (85%)	1 (5%)	0 (0%)	
Coarse-grain cereals, oatmeal, whole-grain pasta	POL	1 (5%)	7 (35%)	5 (25%)	7 (35%)	0 (0%)	0 (0%)	ns
	CZ	1 (5%)	4 (20%)	3 (15%)	11 (55%)	1 (5%)	0 (0%)	
Lard	POL	20(100%)	0 (0%)	0 (0%)	0 (0%)	0 (0%)	0 (0%)	ns
	CZ	19 (95%)	0 (0%)	0 (0%)	1 (5%)	0 (0%)	0 (0%)	
Oils/margarines/butter–margarine mixes	POL	2 (10%)	5 (25%)	1 (5%)	3 (15%)	8 (40%)	1 (5%)	ns
	CZ	4 (20%)	2 (10%)	3 (15%)	7 (35%)	2 (10%)	2 (10%)	
Eggs	POL	1 (5%)	1 (5%)	1 (5%)	13 (65%)	3 (15%)	1 (5%)	ns
	CZ	0 (0%)	0 (0%)	3 (15%)	8 (40%)	6 (30%)	3 (15%)	
Legume seed dishes	POL	2 (10%)	12 (60%)	4 (20%)	2 (10%)	0 (0%)	0 (0%)	ns
	CZ	2 (10%)	8 (40%)	8 (40%)	2 (10%)	0 (0%)	0 (0%)	
Potatoes	POL	0 (0%)	4 (20%)	8 (40%)	8 (40%)	0 (0%)	0 (0%)	ns
	CZ	0 (0%)	4 (20%)	9 (45%)	8 (40%)	0 (0%)	0 (0%)	
Fruit	POL	0 (0%)	0 (0%)	1 (5%)	10 (50%)	3 (15%)	6 (30%)	ns
	CZ	0 (0%)	0 (0%)	0 (0%)	12 (60%)	6 (30%)	2 (10%)	
Vegetables	POL	0 (0%)	0 (0%)	0 (0%)	6 (30%)	5 (25%)	9 (45%)	ns
	CZ	0 (0%)	0 (0%)	1 (5%)	6 (30%)	6 (30%)	7 (35%)	

ns—not statistically significant.

**Table 5:** Frequency of dairy product consumption by female athletes of both study groups (POL—Polish team, CZ—Czech team).

Products	Club	Frequency of Consumption N (%)						*p* Value
		Never	1–3 Times per Month	Once a Week	Several Times a Week	Once a Day	Several Times a Day	
Butter	POL	1 (5%)	5 (25%)	2 (10%)	4 (20%)	6 (30%)	2 10%)	ns
	CZ	3 (15%)	1 (5%)	5 (25%)	8 (40%)	2 (10%)	1 (5%)	
Milk	POL	0 (0%)	1 (5%)	2 (10%)	12 (60%)	4 (20%)	1 (5%)	ns
	CZ	1 (5%)	0 (0%)	3 (15%)	8 (40%)	2 (10%)	6 (30%)	
Fermented milk beverages	POL	0 (0%)	4 (20%)	5 (25%)	10 (50%)	1 (5%)	0 (0%)	ns
	CZ	1 (5%)	4 (20%)	3 (15%)	10 (50%)	2 (10%)	0 (0%)	
Curd cheeses	POL	2 (10%)	2 (10%)	7 (35%)	8 (40%)	1 (5%)	0 (0%)	ns
	CZ	0 (0%)	6 (30%)	7 (35%)	7 (35%)	0 (0%)	0 (0%)	
Cheeses	POL	0 (0%)	4 (20%)	1 (5%)	13 (65%)	2 (10%)	0 (0%)	ns
	CZ	3 (15%)	2 (10%)	4 (20%)	10 (50%)	1 (5%)	0 (0%)	

ns—not statistically significant.

**Table 6:** Frequency of consumption of meat, fish and processed products by female athletes.

Products	Club	Frequency of Consumption N (%)						*p* Value
		Never	1–3 Times per Month	Once a Week	Several Times a Week	Once a Day	Several Times a Day	
Fast-food	POL	0 (0%)	14 (70%)	6 (30%)	0 (0%)	0 (0%)	0 (0%)	ns
	CZ	0 (0%)	15 (75%)	5 (25%)	0 (0%)	0 (0%)	0 (0%)	
Fried foods	POL	0 (0%)	4 (20%)	3 (15%)	11 (55%)	2 (10%)	0 (0%)	ns
	CZ	0 (0%)	8 (40%)	3 (15%)	9 (45%)	0 (0%)	0 (0%)	
Cold cuts, sausages, wieners	POL	2 (10%)	5 (25%)	2 (10%)	7 (35%)	2 (10%)	2 (10%)	ns
	CZ	1 (5%)	8 (40%)	6 (30%)	4 (20%)	1 (5%)	0 (0%)	
Red meat dishes	POL	3 (15%)	8 (40%)	4 (20%)	5 (25%)	0 (0%)	0 (0%)	ns
	CZ	5 (25%)	5 (25%)	9 (45%)	1 (5%)	0 (0%)	0 (0%)	
White meat dishes	POL	0 (0%)	2 (10%)	4 (20%)	11 (55%)	3 (15%)	0 (0%)	ns
	CZ	0 (0%)	2 (10%)	2 (10%)	16 (80%)	0 (0%)	0 (0%)	
Fish	POL	1 (5%)	14 (70%)	5 (25%)	0 (0%)	0 (0%)	0 (0%)	ns
	CZ	0 (0%)	15 (75%)	4 (20%)	1 (5%)	0 (0%)	0 (0%)	
Sweets	POL	0 (0%)	2 (10%)	2 (10%)	16 (80%)	0 (0%)	0 (0%)	ns
	CZ	0 (0%)	1 (5%)	2 (10%)	15 (75%)	2 (10%)	0 (0%)	
Powdered or ready-made soups	POL	7 (35%)	12 (60%)	1 (5%)	0 (0%)	0 (0%)	0 (0%)	ns
	CZ	10 (50%)	7 (35%)	2 (10%)	1 (5%)	0 (0%)	0 (0%)	
Meat preserves	POL	16 (80%)	4 (20%)	0 (0%)	0 (0%)	0 (0%)	0 (0%)	ns
	CZ	19 (95%)	1 (5%)	0 (0%)	0 (0%)	0 (0%)	0 (0%)	
Canned vegetables	POL	8 (40%)	9 (45%)	2 (10%)	1 (5%)	0 (0%)	0 (0%)	ns
	CZ	13 (65%)	6 (30%)	0 (0%)	1 (5%)	0 (0%)	0 (0%)	

ns—not statistically significant.

**Table 7:** Frequency of consumption of selected beverages by female athletes of both study groups (POL—Polish team, CZ—Czech team).

Products	Club	Frequency of Consumption N (%)						*p* Value
		Never	1–3 Times per Month	Once a Week	Several Times a Week	Once a Day	Several Times a Day	
Fruit juices	POL	0 (0%)	5 (25%)	7 (35%)	7 (35%)	1 (5%)	0 (0%)	ns
	CZ	1 (5%)	10 (50%)	5 (25%)	4 (20%)	0 (0%)	0 (0%)	
Vegetable, vegetable and fruit juices	POL	10 (50%)	8 (40%)	0 (0%)	1 (5%)	1 (5%)	0 (0%)	ns
	CZ	8 (40%)	9 (45%)	1 (5%)	2 (10%)	0 (0%)	0 (0%)	
Sweetened hot drinks	POL	3 (15%)	1 (5%)	1 (5%)	5 (25%)	9 (45%)	1 (5%)	ns
	CZ	3 (15%)	3 (15%)	1 (5%)	7 (35%)	4 (20%)	2 (10%)	
Sweetened carbonated and non-carbonated drinks	POL	3 (15%)	5 (25%)	4 (20%)	7 (35%)	1 (5%)	0 (0%)	ns
	CZ	1 (5%)	8 (40%)	6 (30%)	5 (25%)	0 (0%)	0 (0%)	
Energy drinks	POL	9 (45%)	5 (25%)	2 (10%)	3 (15%)	1 (5%)	0 (0%)	ns
	CZ	11 (55%)	4 (20%)	2 (10%)	2 (10%)	1 (5%)	0 (0%)	
Water	POL	0 (0%)	0 (0%)	0 (0%)	0 (0%)	1 (5%)	19 (95%)	ns
	CZ	0 (0%)	3 (15%)	0 (0%)	2 (10%)	1 (5%)	14 (70%)	
Isotonic drinks	POL	7 (35%)	7 (35%)	2 (10%)	4 (20%)	0 (0%)	0 (0%)	ns
	CZ	3 (15%)	10 (50%)	1 (5%)	6 (30%)	0 (0%)	0 (0%)	
Alcoholic beverages	POL	7 (35%)	9 (45%)	4 (20%)	0 (0%)	0 (0%)	0 (0%)	
	CZ	4 (20%)	15 (75%)	1 (5%)	0 (0%)	0 (0%)	0 (0%)	ns

ns—not statistically significant.

## Data Availability

Data are available upon request due to restrictions of privacy or ethics.
